# Fully tunable Fabry-Pérot cavity based on MEMS Sagnac loop reflector with ultra-low static power consumption

**DOI:** 10.1038/s41378-024-00728-y

**Published:** 2024-08-29

**Authors:** Young Jae Park, Man Jae Her, Youngjae Jeong, Dong Ju Choi, Dong Uk Kim, Min Gi Lim, Myung Seok Hong, Hyug Su Kwon, Kyoungsik Yu, Sangyoon Han

**Affiliations:** 1 Department of Robotics and Mechatronics Engineering, Daegu Gyeongbuk Institute of Science and Technology (DGIST), Daegu, 42988 Republic of Korea; 2 School of Electrical Engineering, Korea Advanced Institute of Science and Technology (KAIST), Daejeon, 34141 Republic of Korea; 3 Agency for Defense Development, Daejeon, Republic of Korea

**Keywords:** Engineering, NEMS, Applied optics, Electrical and electronic engineering

## Abstract

The Fabry-Pérot interferometer, a fundamental component in optoelectronic systems, offers interesting applications such as sensors, lasers, and filters. In this work, we show a tunable Fabry-Pérot cavity consisting of tunable Sagnac loop reflectors (SLRs) and phase shifters based on electrostatic microelectromechanical (MEMS) actuator. The fabrication process of the device is compatible with the standard wafer-level silicon photonics fabrication processes. This electrostatic actuation mechanism provides well-balanced, scalable pathways for efficient tuning methodologies. The extinction ratio of the continuously tunable SLRs’ reflectivity is larger than 20 dB. Full 2π phase shifting is achieved, and response times of all the components are less than 25 μs. Both actuators have extremely low static power, measuring under 20 fW and the energy needed for tuning is both below 20 pJ.

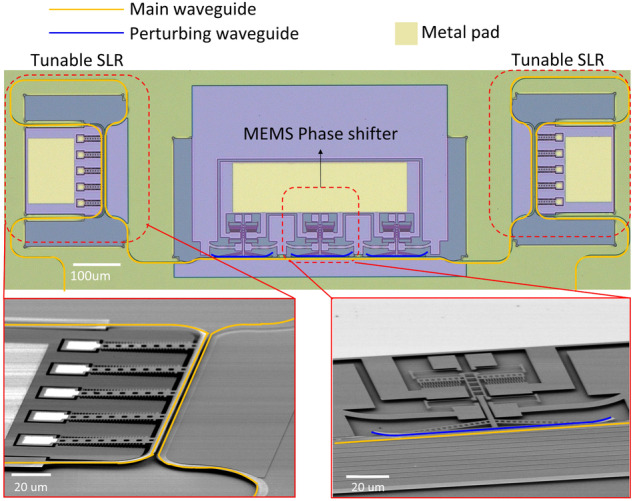

## Introduction

The field of silicon photonics is experiencing rapid growth, primarily due to its compatibility with complementary metal-oxide-semiconductor (CMOS) manufacturing technology^1,2^. The silicon photonics technology offers significant advantages over free space or fiber-based technologies in terms of cost, complexity, speed, energy consumption, etc. As a result, a variety of high-performance optical devices and systems have been implemented and manufactured in the integrated form.

One of the fundamental components in optoelectronic systems is the Fabry-Pérot interferometer^3^. Considerable efforts have been made to implement this component on silicon photonics platforms so far. The integrated Fabry-Pérot interferometer finds its applications in optical sensors^4–9^, lasers^10–14^, and filters^15–18^. While many on-chip Fabry-Pérot interferometers have been developed based on Bragg gratings^19–22^, they often require complex design efforts and delicate fabrication processes due to their subwavelength scale sizes. Moreover, real-time tuning of their reflection characteristics is difficult or often time impossible.

In contrast, Sagnac loop reflector (SLR) offers a promising alternative to Bragg gratings due to their simple structure and straightforward working mechanism. Several integrated SLRs with tunable reflectivity have been designed using a Mach-Zehnder interferometer (MZI)-based thermo-optic methodology^23–26^. However, the tunable SLRs have drawbacks including high electrical power consumption and thermal crosstalk, making them less suitable for large-scale and high-density applications. Additionally, the MZI-based designs introduce complexity by requiring two directional couplers and a thermal phase shifter per unit element.

Micro-electromechanical systems (MEMS) present a viable solution to address these limitations. Electrostatic MEMS technology requires low electrical energy consumption for tuning and extremely low electrical power consumption for maintaining specific states. Importantly, it does not generate heat and therefore, eliminates crosstalk between unit devices, facilitating high-density integration. Numerous works have successfully demonstrated energy-efficient optical devices and systems using MEMS, demonstrating its feasibility in integrated photonics^27–32^. However, so far, the MEMS-based approaches have been limited to either optical switching devices^33^ or standalone phase shifters^28^.

We created a Fabry-Pérot using a Sagnac loop before^34^. However, at that time, we did not have a phase shifter, preventing us from fully tuning the Fabry-Pérot cavity. Subsequently, we added a MEMS-tunable phase shifter to create the Fabry-Pérot cavity^35^. However, during this time, there was an issue where the coupler was not perfectly lifted in the vertical out-of-plane direction, causing reflectivity even without applying voltage.

In this research, we present an integrated fully tunable Fabry-Pérot cavity based on MEMS-tunable SLRs and MEMS-tunable phase shifters, enabling precise control over transmissivity, reflectivity, and the resonance frequency of the cavity. We believe our work will broaden the scope of the applications of MEMS methodology in integrated optics.

## Results

### Fully tunable Fabry-Pérot cavity overview

**Fig. 1:**
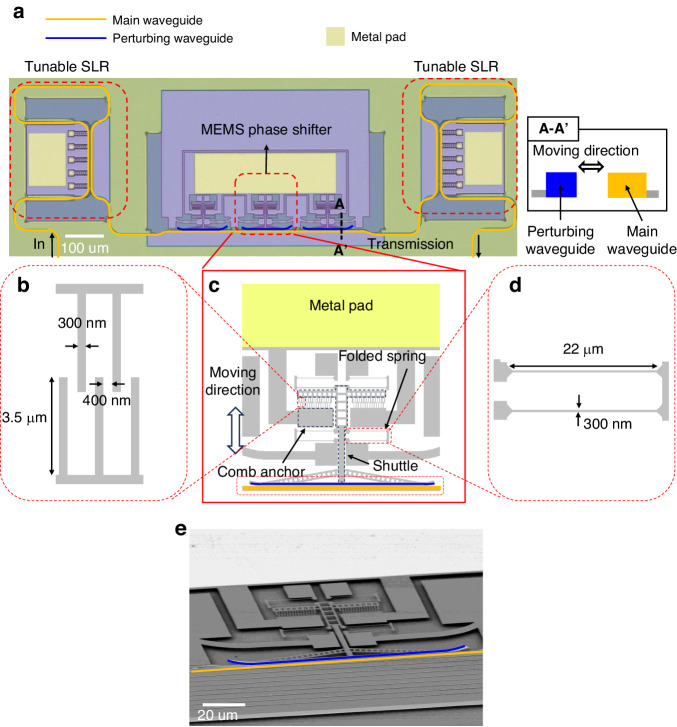
Schematic and microscope images of the Fabry-Pérot cavity. **a** Optical microscope image of the Fabry-Pérot cavity. **b** Layout of the comb-fingers. **c** Layout of the MEMS-tunable phase shifter. **d** Layout of the folded-beam spring. **e** SEM image of the MEMS-tunable phase shifter

Figure 1a depicts the optical microscope image of our fully tunable Fabry-Pérot device. Our device consists of two interconnected MEMS-tunable SLRs positioned facing each other. The MEMS-tunable phase shifters, designed for tuning the resonant wavelength of the Fabry-Pérot cavity lie between the two SLRs. We used three of the phase shifters in series to have an extra phase shift beyond 2π. The yellow and the blue lines highlighted in the picture are a main waveguide which guides light, and a movable perturbing waveguide of the phase shifters, respectively. The footprint of the fully tunable Fabry-Pérot device measures less than 0.53 mm². The device was fabricated on a 200 mm silicon-on-insulator (SOI) wafer with CMOS-compatible processes. The details of the fabrication are described in “Materials and methods” section.

### MEMS-tunable phase shifter

**Fig. 2:**
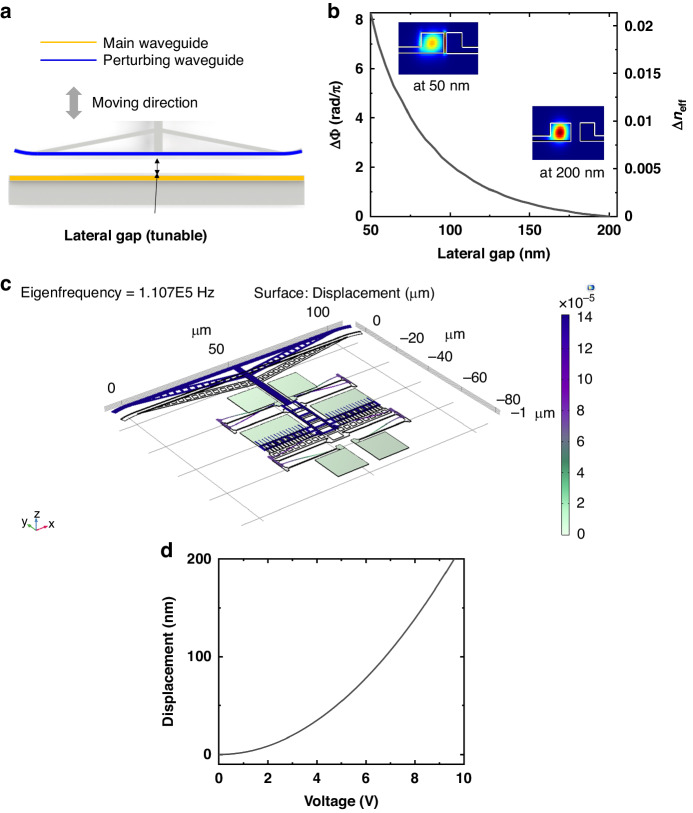
Working principle, simulation and calculation results of the MEMS-tunable phase shifter. **a** Working principle of the MEMS-tunable phase shifter. **b** Simulated optical response of the MEMS-tunable phase shifter versus lateral gap (insets: mode profiles at 50 and 200 nm lateral gaps). **c** Simulated resonance frequency of MEMS-tunable phase shifter. **d** Calculated voltage versus displacement of the lateral comb actuator

Figure 1c shows the detailed schematic of the phase shifter device. The working principle of the device is similar to ref. ^27^. We employed a lateral comb-drive actuator to move the perturbing waveguide. The perturbing waveguide is connected to the actuator via the shuttle, allowing horizontal motion. The main waveguide and the perturbing waveguide have widths of 450 and 300 nm, respectively. The initial gap between the two waveguides is 200 nm. The phase shifter is 100 µm-long, and capable of inducing a full 2π-phase shift. The three-phase shifters are associated with a single metal pad, ensuring uniform movement when voltage is applied. The dimension of the metal pad for electrical probing and wire bonding is a total of 100 × 350 µm² for the three-phase shifters. Figure 1b provides a detailed view of the comb-fingers. There are two sets of comb-fingers: one attached to the stationary anchors and the other to the shuttle. The comb-fingers have a width of 300 nm, a length of 3.5 μm, and the nearest distance between the comb-fingers is 400 nm. When a voltage difference is applied between the two sets of the comb fingers, the electric force between the sets pulls the shuttle forward. There are four folded-beam springs that hold the shuttle, which balance the electrical forces between the comb-finger sets to maintain the location of the shuttle. Figure 1d shows the details of the folded-beam spring. The spring has a width of 300 nm and a length of 22 μm. The scanning electron microscope (SEM) image of the phase shifter is shown in Fig. 1e. Figure 2a, b provides a detailed view of the optical operating principle of the phase shifters and the simulated result obtained through Lumerical simulation. When a voltage is applied to the actuator, the perturbing waveguide (Fig. 2a blue line) approaches the main one (Fig. 2a yellow line), causing the optical mode in the main waveguide to expand towards it (Fig. 2b inset, at 50 nm), increasing the mode’s effective refractive index (Fig. 2b). The amount of phase shifts ($\Delta \phi$) is calculated from the effective refractive index using the equation below.

1 $$\Delta \phi =\frac{2\pi \times L\times {\varDelta n}_{{eff}}}{\lambda }$$

where $L$, $\varDelta {n}_{{eff}}$, $\lambda$, and $\Delta \phi$ are the length of the perturbing waveguide, the amount of effective index change, the operating wavelength, and the amount of phase shift, respectively. When the gap changes from 200 nm to 100 nm, the effective index of the mode changes by 0.005, and a full 2π-phase shift is achieved. Figure 2c illustrates the simulated resonant frequency of the MEMS-tunable phase shifter. The resonant frequency obtained through COMSOL simulation is 110 kHz.

The calculated response of the MEMS-tunable phase shifter is shown in Fig. 2d. The relationship between the displacement and the applied voltage of the lateral comb-drive actuator can be calculated using the equation below^36^.

2 $$V=\sqrt{\frac{{kgx}}{{\varepsilon }_{0}{tn}}}$$

where $k$, $x$, $n$, ${\varepsilon }_{0}$, $V$, $t$ and $g$ are spring constant of spring, displacement, number of comb fingers, permittivity of air, voltage applied to the comb-fingers, thickness of comb-fingers, and the gap between comb-fingers, respectively. The voltage required to move 200 nm is 9.6 V, where $k=0.1674 {{N}/m}$, $g=300{\rm{nm}},{x}=200{\rm{nm}},\,{\varepsilon }_{0}=8.85\times {10}^{-12}{{F}/m}$, $t=220{{nm}},{n}=56$.

### MEMS-tunable Sagnac loop reflector

**Fig. 3:**
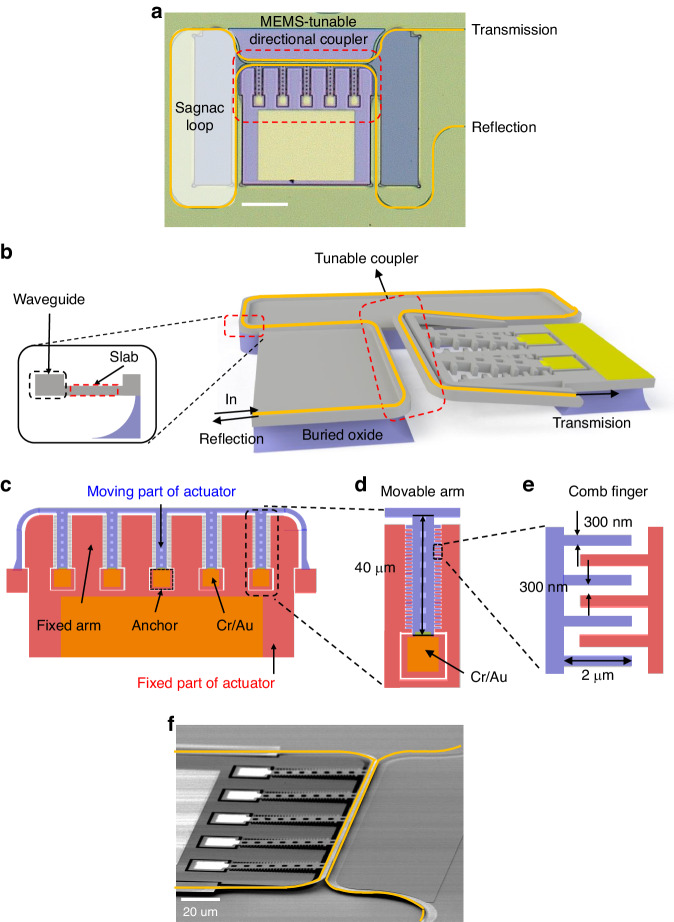
Schematic and microscope images of the Sagnac loop reflector. **a** Optical microscope image of the Sagnac loop reflector. **b** 3D schematic of the Sagnac loop reflector. **c** Layout of the vertical comb-drive actuator. **d** Layout of the movable arm. **e** Layout of the comb-fingers. **f** SEM image of the MEMS-tunable directional coupler

Figure 3a shows the optical microscope image of our standalone tunable SLR device, comprised of a MEMS-tunable directional coupler and a Sagnac loop. The yellow lines in the figure represent the waveguides through which light propagates. The total device area measures less than 0.09 mm², and the dimension of the metal pad for electrical probing and wire bonding is 90 × 130 µm². In Fig. 3b, a 3D schematic of the device is presented. The waveguide has a thickness of 220 nm and a width of 450 nm, and it features a 70 nm-thick slab on one side to provide mechanical support (as depicted in Fig. 3b inset). These waveguide dimensions are designed for operation at wavelengths around 1550 nm in the transverse electric (TE) mode, with effective and group indices of 2.33 and 4.38, respectively. Figure 3c shows the layout of the vertical comb-drive actuator^37^ that constitutes the MEMS-tunable directional coupler. The actuator consists of two main components: a movable part responding to an applied electric field (marked in blue), and a stationary part (marked in red). The movable part comprises five movable arms of which ends are attached to anchors (Fig. 3d), with Cr/Au films on the anchor to introduce tensile stresses, causing the movable arms to vertically elevate by 1.5 µm. The length of the movable arm is 40μm. By applying a voltage difference between the comb-fingers at the fixed part and the movable part, we can precisely control the vertical offset of the movable part, and consequently, we can manipulate the state of the tunable coupler. Figure 3e shows the dimension of the comb-fingers. The width of the comb finger is 300 nm and the lateral gap between the nearest comb fingers when the movable comb fingers align with the static comb fingers in the same plane (i.e., vertical offset = 0 nm) is 300 nm. The length of the comb finger is 2 µm. The gap between the comb fingers is designed based on the foundry’s process experience, using proven gap where successive lithography result is proven. Also, the length of the comb finger is designed considering sufficient stiffness to prevent stiction caused by electrostatic force. The scanning electron microscope (SEM) image of the tunable coupler is shown in Fig. 3f.

**Fig. 4:**
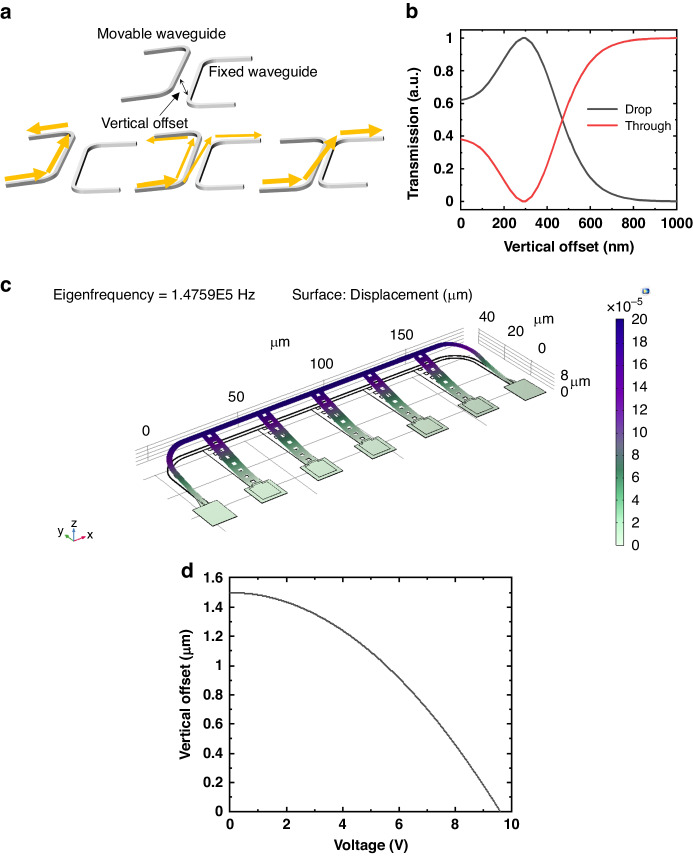
Working principle, simulation and calculation results of the MEMS-tunable directional coupler. **a** Working principle of the MEMS-tunable directional coupler. **b** Simulated optical response of the MEMS-tunable directional coupler versus vertical offset. **c** Simulated natural frequency of the MEMS-tunable directional coupler. **d** Calculated response of the MEMS-tunable directional coupler

Figure 4a describes the working principle of the tunable directional coupler. The coupler comprises two parallel waveguides, one of which is attached to the tips of the movable arms. By adjusting the vertical offset of the movable arms, we can change the vertical position of the waveguide connected to the tips, thereby enabling us to manipulate the coupling strength between the two waveguides. Consequently, the splitting ratio of the directional coupler changes. The directional coupler’s straight coupling section is 150 µm in length. When the two waveguides align within the same plane (i.e., vertical offset = 0 nm), the lateral gap between them is 200 nm. The simulated optical response of the tunable directional coupler is plotted in Fig. 4b. As the vertical offset changes from 1000 nm to 300 nm, the coupling ratio changes from 0 to 1. Figure 4c shows the simulated resonance frequency of our MEMS-tunable directional coupler. The resonance frequency obtained through COMSOL simulation for vertical comb actuator is 147.6 kHz. Figure 4d shows the calculated response of the MEMS-tunable directional coupler. To analytically calculate the relationship between the applied voltage and vertical offset of the directional coupler, we make some assumptions about the actuator.

- The length of the deposited metal on the movable arm is very short compared to the silicon arm. Therefore, we ignored the effect of the metal.
- The comb fingers of the actuator are arranged at regular intervals. Therefore, we approximate the force acting between the comb fingers as a uniformly distributed load for calculation.
- We assumed that the change in capacitance in the z-direction remains constant even as the height of the actuator changes.

The deflection of the beam under the uniformly distributed load *w* is defined by the following equation^37^.

3 $$\delta =\frac{w{l}^{4}}{8{EI}}$$

where $E$ is the Young’s modulus of the silicon, $I$ is the moment of inertia of the movable arm, and $l$ is the length of the movable arm. The tip of the movable arm of the actuator is lifted by a total of 1.5 μm. If this beam is pulled by 1.5 μm (${\delta }_{\max }$ = 1.5 μm), it can be considered that the movable arm is pulled to its maximum extent. At this point, the voltage required is the maximum voltage for the actuator.

To calculate the uniform distributed load $w$, we need to utilize the electrical force equation for vertical comb fingers pair. The equation is as follows^38^.

4 $${F}_{E}=\frac{1}{2}\cdot N\cdot {L}_{c}\cdot \frac{{dC}}{{dZ}}\cdot {V}^{2}$$

Where $N$ is the number of the comb fingers pair, ${L}_{c}$ is the overlap length of the comb fingers, $\frac{{dC}}{{dZ}}$ is the gradient of capacitance of comb finger in the z direction and $V$ is the applied voltage. Since there are two comb fingers pairs per 1.2 μm, reflecting this, the equation for deflection of movable arm under uniformly distributed load can be written as follows:

5 $$\delta =\frac{N\cdot {L}_{c}\cdot \frac{{dC}}{{dZ}}\cdot {V}^{2}{\cdot l}^{4}}{16\cdot E\cdot I\cdot 1.2}$$

And by subtracting $\delta$ at 1.5 μm, we can determine the vertical offset of the movable arm according to the applied voltage. The graph of the calculated response is shown in Fig. 4d. The related simulation parameters are presented in Table 1.

**Table 1:** The parameter table of simulation

Symbol	Value	Symbol	Value
$N$	2	$E$	150 [Gpa]
${L}_{c}$	1.5 [μm]	$\frac{{dC}}{{dZ}}$	26.5 [pF/m]
$l$	43.75 [μm]		

### Optical and electrical measurement

All experiments were conducted under ambient air conditions.

**Fig. 5:**
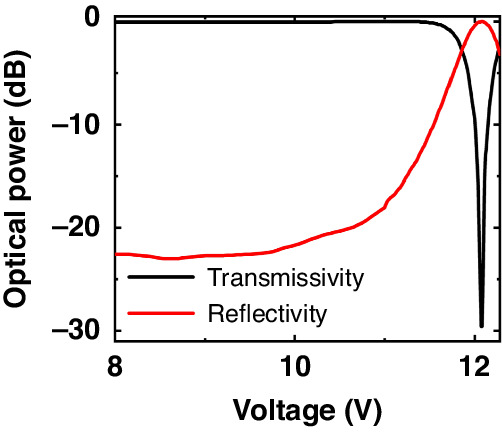
Measured optical responses of the Sagnac loop reflector in response to the applied voltage

**Fig. 6:**
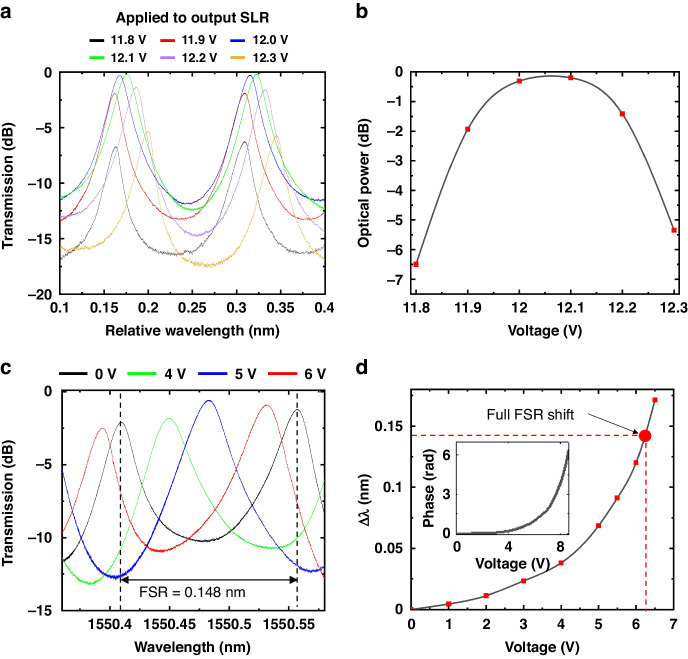
Measured optical responses of the Fabry-Pérot cavity. **a** Measured spectral response of the Fabry-Pérot cavity with various voltage applied to the output SLR. **b** Transmitted optical power of the Fabry-Pérot cavity at its resonance versus voltages applied to the output mirror. **c** Measured spectral responses of the Fabry-Pérot cavity with various voltages applied to the phase shifter. **d** Measured resonance shift of the Fabry-Pérot cavity versus voltage applied to the phase shifter (Inset = measured resonance shift of the single MEMS-tunable phase shifter)

Figure 5 shows the measured optical response of the SLR with various voltages applied to the tunable directional coupler. By adjusting the voltage applied to the coupler, the coupling ratio of the coupler changed, and as a result, both the transmissivity and the reflectivity of the SLR were changed. At 12.1 V, the transmissivity reached its minimum while the reflectivity reached its maximum, resulting in extinction ratios as high as 29.2, and 23.7 dB for transmissivity and reflectivity, respectively. The transmissivity of the Fabry-Pérot cavity at its resonant wavelengths can be described as the equation below^39^.

6 $${T}_{{resonance}}={\left[\frac{{I}_{{out}}}{{I}_{{in}}}\right]}_{{resonance}}=\frac{\left(1-{R}_{{in}}\right)\left(1-{R}_{{out}}\right)}{{\left(1-\sqrt{{R}_{{in}}{R}_{{out}}}\right)}^{2}}$$

where ${I}_{{in}}$, ${I}_{{out}}$, ${R}_{{in}}$, ${R}_{{out}}$ are the intensity of input and output at the resonance, reflectivity of the input mirror, and reflectivity of the output mirror, respectively. From Eq. (6) we can derive that the transmissivity at the resonance wavelength is maximized when ${R}_{{in}}={R}_{{out}}$. In Fig. 6a, we present the transmissivity spectrum of the Fabry-Pérot cavity with different voltages (11.8, 11.9, 12.0, 12.1, 12.2, and 12.3 V) applied to the SLR located at the output side of the cavity. Meanwhile the reflectivity of the input SLR was fixed near 0.1. As shown in the figure, the transmissivity spectrum of the cavity changes as we change the voltage applied to the output SLR. We plotted the transmissivity of the cavity at its resonant wavelength versus the applied voltage to the output SLR in Fig. 6b. As shown in the plot, the transmissivity was maximized when 12.1 V was applied to the output SLR. At this voltage, the reflectivity of the output SLR becomes 0.1 (see Fig. 5), matching the reflectivity we set for the input SLR. Therefore, we can confirm that our Fabry-Pérot cavity aligns well with Eq. (2), showing a regular behavior of Fabry-Pérot cavity. There is a discrepancy between the theoretical calculation and measurement of the voltage at which the coupling occurs. This is because the capacitance does not change linearly as the deflection of the movable arm reaches its maximum, causing this difference. Additionally, due to process tolerances, the actual width of the comb may become thinner, resulting in a smaller capacitance compared to the simulation. In Fig. 6a, we noted that the resonance wavelength of the cavity slightly redshifts as we increase the voltage applied to the output SLR. We attribute this phenomenon to the coupling-induced resonance frequency shifts (CIFS) effect^40^. As the voltage applied to the SLR increases, the movable waveguide in its tunable directional coupler approaches the fixed waveguide, and as a result, CIFS occurs. Figure 6c demonstrates the measured spectrum shift as a function of the voltage applied to the phase shifters. The resonance peak redshifts as the voltage applied to the phase shifters increases. As shown in Fig. 6d, the resonance of the cavity shifts by its full FSR by applying more than 6.5 V to the phase shifters. The phase shifter response that measured in test structure is shown in Fig. 6d inset. By applying over 8.5 V, the full 1 FSR shifts is achieved. We measured the insertion loss of the phase shifters using a test structure. The measured insertion loss was 0.36 dB at 0 phase shift and 0.39 dB at a 2π phase shift. The value of modulation efficiency ${V}_{\pi }{L}_{\pi }$ is 0.00153.

### Electrical power and energy consumption

**Fig. 7:**
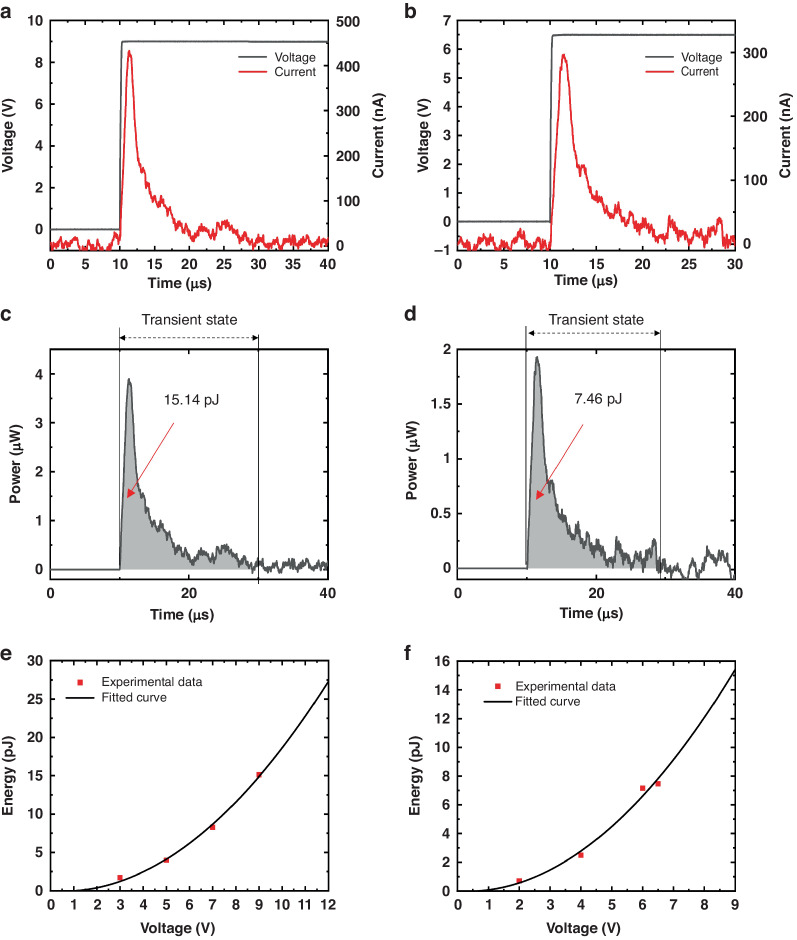
Measured power and energy consumption of MEMS actuators. Measured current responses of the MEMS-tunable directional coupler (**a**) and the MEMS-tunable phase shifter (**b**). Electrical power consumption of the MEMS-tunable directional coupler (**c**) and the MEMS-tunable phase shifter (**d**) calculated from (**a**), (**b**), respectively. Electrical energy consumptions of the MEMS-tunable directional coupler (**e**) and the MEMS-tunable phase shifter (**f**) versus applied voltages

Figure 7a, b presents the measured current flows of the MEMS-tunable coupler and phase shifters, respectively, while applying step voltages to the devices. To calculate the electrical power consumption, we multiplied the measured current with the applied voltages, yielding the power consumption graphs for the MEMS-tunable directional coupler and phase shifters, shown in Fig. 7c, d respectively. As shown in the graphs, the electrical power consumptions during the transient times were a few micro-watts for both devices. These values are more than 1000 times less than the conventional thermo-optic devices. By integrating the area under the power consumption graphs, we calculated the total electrical energy required to tune the devices to certain voltages as plotted in Fig. 7e, f. As shown in the graphs, the electrical energies required to maximally tune the tunable coupler and the phase shifters are less than 30 and 16 pJ, respectively. We also attempted to measure the static electrical power consumption by measuring the static current flows of the devices while maintaining the states of the devices after the transient times. However, the electrical currents at static state were less than the minimum measurable value (75 nA) of the current meter we used. So, we use module (4200-pa preamp, Keithley) that is suitable for measuring static power. At steady state, the electrical power consumption of the tunable directional coupler and phase shifters is less than 10 fW and 15 fW, respectively, when achieving 100% reflectivity and a 2pi phase shift.

### Response time measurement

**Fig. 8:**
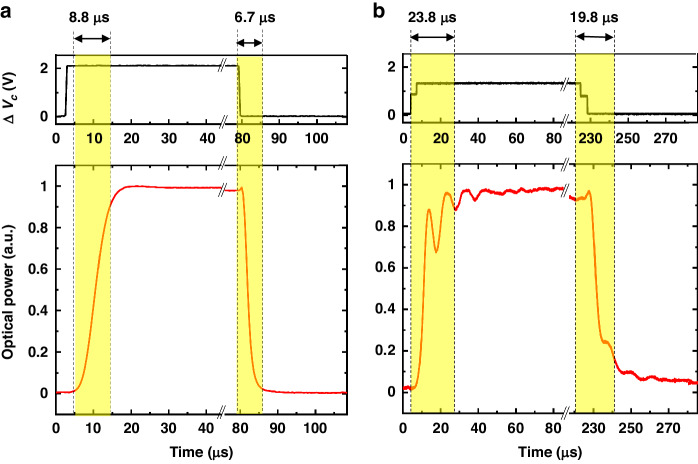
Measured time response of MEMS actuators. Measured time response of the MEMS-tunable directional coupler (**a**) and the MEMS-tunable phase shifter (**b**)

Figure 8a, b illustrates the measured time responses of the tunable coupler and phase shifters. The rise and fall times are defined as the durations required to reach 90% and 10% of the maximum optical power, respectively. Specifically, the rise time and fall time of the tunable coupler were measured at 8.8 and 6.7 μs, respectively, while the phase shifters exhibited rise and fall times of 23.8 and 19.8 μs, respectively. The simulated time response of the tunable coupler and phase shifter is 9 μs and 6.8 μs. The simulated time response of the phase shifter is quite different from measured data. We conclude that this is influenced by the area related with the movement direction. It moves in-plane direction with a small cross-sectional area, resulting minimum air damping effects. As a result, ringing occurs, causing an extended time response. Furthermore, despite the fabrication of three identical structures of the phase shifter, process variance introduces slight differences among them. These variations in the manufacturing process contribute to disparities in the response characteristics, potentially leading to ringing. Their values are comparable to that of thermo-optic-based devices.

## Discussion

We have successfully designed and experimentally demonstrated the tunable Fabry-Pérot cavity having ultra-low electrical power consumption by utilizing the MEMS-tunable SLR and the MEMS-tunable phase shifter. The transmissivity and the resonance wavelength of the cavity can be fully tuned with reconfiguration times less than 25 μs. The MEMS tuning elements consume only tens of pJ levels of energy for reconfiguration and consume less than 675 nW for maintaining their states. Ideally, there should be no static power consumption in our MEMS-tunable devices as their actuation mechanism is capacitive. However, there are finite leakage currents through the BOX layer between the metal pads of the actuators and the silicon substrate. We can estimate the static power consumption more accurately by calculating the leakage current using the resistivity of SiO_2_ (i.e., BOX). The resistivity of BOX is known as 10^16^ Ω·cm at ref. ^41^. The resistances of the BOX between the devices and the silicon substrate can be calculated using the well-known equation below.

7 $$R=\rho \frac{l}{A}$$

where $\rho ,{l},\,{\rm{and}}{A}$ are the resistivity, thickness, and area of the BOX below the devices, respectively. The tunable coupler and the phase shifters each have a metal pad situated above the 2 μm-thick BOX layer. The area of the BOX beneath the tunable coupler’s metal pad is 21,300 μm^2^, leading to a resistance of 9.39 × 10^15^ Ω between the pad and the silicon substrate. Similarly, for the phase shifters, the BOX area under the device is 34,100 μm^2^, resulting in a resistance of 5.87 × 10^15^ Ω. From the calculated resistances, we can calculate the static power consumptions due to the leakage currents using Ohm’s law. Therefore, the static power consumptions of the tunable coupler and the phase shifters are 10.6 fW (for 10 V) and 7.2 fW (for 6.5 V), respectively. These values are several orders of magnitude less than the power consumption of thermo-optic-based tuners (i.e., tens of milliwatts). And also the order of the magnitude is similar to the measured data. Therefore, our devices can introduce tuning functions to various applications that are sensitive to heat. For example, our device concept can be used to add flexible tunability to the integrated lasers without degrading the performance of the lasers. Also, a silicon-based MEMS actuator can actuate in the low temperature condition. The research that actuates silicon MEMS mirror at cryogenic conditions is reported^42^. So, we consider that the novel device concept presented in this paper will provide unrestricted tunability to device that have previously been limited in tunability due to their sensitivity to temperature or low electrical power budgets.

## Materials and methods

### Device fabrication

The devices were fabricated at the National NanoFab Center (NNFC) in South Korea, following a process closely resembling the standard silicon photonics platform, incorporating a KrF scanner. The devices were fabricated on an 8-inch silicon-on-insulator (SOI) wafer with a 220 nm-thick silicon device layer and a 2 μm-thick buried oxide (BOX) layer deposited using LPCVD. At the beginning of the fabrication process, a 100 nm-thick SiO_2_ layer was deposited on the top of the wafer, serving as a hard mask. Subsequently, a sequence of three lithography steps using I-line stepper was applied to the wafer, each followed by silicon dry etching processes with depths of 70 nm, 80 nm, and 70 nm, respectively. Following the last dry etching process, an additional lithography step was introduced to execute a metal lift-off process. During this stage, layers of Chromium (Cr) and Gold (Au) were consecutively deposited onto the wafer utilizing an electron beam evaporation process. These metallic films possessed thicknesses of 5 nm for Cr and 50 nm for Au, with lift-off accomplished using Acetone. In the final stage, a vapor hydrofluoric acid etching process was employed to selectively etch the buried oxide layer, enabling the release and mobility of the actuators and waveguides.

### Device characterization

For device characterization, we used an electrical/optical measurement setup equipped with electrical probes and an optical fiber array. The chip was securely positioned on a metal plate, establishing electrical grounding through the plate and consequently, grounding the chip’s substrate. To apply voltages to the devices, we utilized a combination of electrical power supplies (Gwinstek GPP-4323) and function generators (Keysight 33600A) connected to the electrical probes. The current flow through the devices was measured using a current meter (Keithley 4200A). We used a tunable diode laser (CTL 1550, Toptica), an optical power meter (PM100D, Thorlabs), and a photodetector (1811-FC, Newport) for optical characterization. A 48-channel fiber array was used to optically access grating couplers connected to the device.
